# Biorenewable Deep Eutectic Solvent for Selective and Scalable Conversion of Furfural into Cyclopentenone Derivatives

**DOI:** 10.3390/molecules23081891

**Published:** 2018-07-28

**Authors:** Maria Luisa Di Gioia, Monica Nardi, Paola Costanzo, Antonio De Nino, Loredana Maiuolo, Manuela Oliverio, Antonio Procopio

**Affiliations:** 1 Department of Pharmacy, Health and Nutritional Sciences, University of Calabria, Arcavacata di Rende, 87036 Cosenza, Italy; ml.digioia@unical.it; 2 Department of Chemistry and Chemical Technologies of the University of Calabria, Cubo 12C, Arcavacata di Rende, 87036 Cosenza, Italy; antonio.denino@unical.it (A.D.N.); loredana.maiuolo@unical.it (L.M.); 3 Agrarian Department, San Raffaele Telematic University, Via di Val Cannuta, 247, 00166 Roma, Italy; 4 Department of Health Sciences, Magna Graecia University, Viale Europa, 88100 Germaneto CZ, Italy; pcostanzo@unicz.it (P.C.); m.oliverio@unicz.it (M.O.); procopio@unicz.it (A.P.)

**Keywords:** biomass conversion, cyclopentenones, deep eutectic solvents, furfural, green chemistry

## Abstract

The development of novel synthetic routes to produce bioactive compounds starting from renewable sources has become an important research area in organic and medicinal chemistry. Here, we present a low-cost procedure for the tunable and selective conversion of biomass-produced furfural to cyclopentenone derivatives using a mixture of choline chloride and urea as a biorenewable deep eutectic solvent (DES). The proposed medium is a nontoxic, biodegradable, and could be reused up to four times without any unfavorable effect on the reaction yield. The process is tunable, clean, cheap, simple and scalable and meets most of the criteria; therefore, it can be considered as an environmental sustainable process in a natural reaction medium.

## 1. Introduction

In recent years, green chemistry has gained a great deal of attention, due to its ability to provide new processes that minimize the use or generation of harmful substances, focusing the attention on the use of cheap, renewable, and environmentally safe starting materials. In this regard, bio-based materials have attracted many attentions in the last two decades; therefore, a list of top value added chemicals from biomass has been drafted by the U.S. Department of Energy. Furfural, easily obtained from xylose, is considered in that list as a valuable chemical building block to synthesize other functionalized compounds [1,2,3,4,5,6,7]. One important class of new products derived from furfural are cyclopentenones, which are distinguished structural motifs present not only in notable natural molecules but also versatile as synthetic intermediates.

**Figure 1:**
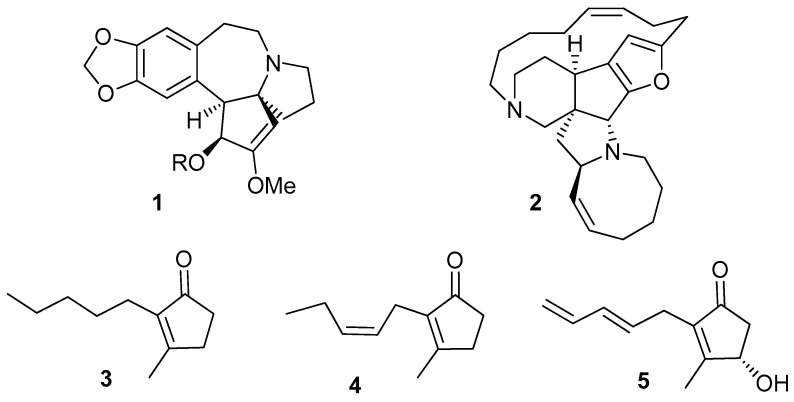
The cyclopentenone moiety into biologically active molecules: (2)-cephalotaxine ester derivatives **1**, (+)-nakadomarin **2**, dihydrojasmones **3**, cis-jasmones **4** and rethrolones **5.**

The introduction of the cyclopentenone moiety into molecules, such as (2)-cephalotaxine ester derivatives **1** and (+)-nakadomarin **2**, has been shown to boost their anticancer potential. Furthermore, this five-membered ring structure is found in many compounds used in the perfumery industry, such as dihydrojasmones **3** and cis-jasmones **4**, as well as in biologically active compounds, including rethrolones **5** employed in the synthesis of pyrethrines, prostanoids, methylenomycins (Figure 1) [8,9,10].

Numerous conventional synthetic approaches reported on the construction of the cyclopentenone framework suffer from disadvantages, such as requirement of expensive catalysts, excess of toxic solvents, harsh reaction conditions, undesirable wastes, unsatisfactory yields, and cumbersome product isolation procedures [11,12,13,14]. Therefore, in the last decade, considerable research has been devoted to the use of catalytic systems, such as Sc(OTf)_3_ and Dy(OTf)_3_, for the synthesis of *trans*-4,5-diaminocyclopent-2-enones [15]. In this context, erbium(III) chloride has found to act as a powerful catalyst to convert biorenewable furfural derivatives into bifunctionalized cyclopentenones in a green solvent, such as ethyl lactate [16], as well as in heterogeneous catalysts [17,18]. 

Within the framework of green chemistry, solvents occupy a strategic place, and in the literature, novel approaches have also been proposed, addressing the use of safer alternative media for this kind of transformation. Ramesh et al. employed an acidic ionic liquid as a reusable catalyst for the reaction of furfural and secondary amines to yield *trans*-4,5-diaminocyclopent-2-enones [19], and our group recently demonstrated the successful use of water as a reaction medium for the preparation of cyclopentenone derivatives under microwave irradiation [20,21]. In addition, a very recent paper from Afonso’s group described the use of Cu(OTf)_2_ as a very efficient catalyst for the synthesis of cyclopentenones in water at room temperature for only 1 min [22]. Water is undoubtedly the cleanest solvent on the Earth, as it is cheap, nontoxic, nonflammable, biorenewable, and safe for humans and the environment [23,24,25,26]. Nevertheless, pure water is precious, and when it is contaminated by chemicals, its purification is difficult and expensive [27], indicating that water can be considered to be a truly green solvent only if it can be directly discharged to a biological effluent treatment plant. Meanwhile, the use of water in various applications is limited. Therefore, the promising approach is to seek more solvents, identify their characteristics that promote the selected process, and then choose a suitable green solvent with required properties. For this purpose, a large set of solvents could be the best alternative to the ideal “solvent-free condition”.

Deep eutectic solvents (DESs) are promising sustainable alternatives to traditional solvents and ionic liquids. They are generally composed of two or three safe and inexpensive components, which are involved in hydrogen bond interactions with each other to obtain a eutectic mixture with a melting point much lower than that of each component species [28]. Since their typical components (e.g., choline chloride (ChCl), urea, glycerol (Gly), natural carboxylic acids, amino acids and carbohydrates, polyalcohols, etc.) come from natural sources, DESs display low toxicity, high thermal stability, high biodegradability, high recyclability, low inflammability and volatility [29,30]. In addition, the preparation of DES is easy and cost-effective and does not involve any post purification or disposal problems [31,32]. DESs have been used for a variety of applications, including metal deposition, metal oxide processing, metal dissolution, extractions and a variety of synthetic processes. New emerging fields of applications of DESs are represented by metal-, bio-, and organocatalysis, and by organometallics [33,34,35,36,37,38,39,40,41]. All of these benefits prompted us to use DESs in our research. Herein, we reported an experimentally and environmentally convenient method for the synthesis of bifunctionalized cyclopentenones from furfural and amines using a biorenewable ChCl-based DES.

## 2. Results and Discussion

Our investigation started with the model reaction between 2-furaldehyde and morpholine in different ChCl-based DESs in order to optimize the reaction conditions. Initially, the effect of these media on the system furfural/morpholine was studied performing the reaction in the DES formed by ChCl and glycerol (Table 1, entries 1 and 2). Although no reaction was observed at room temperature, evidence that the system could work well was registered when the same reaction was performed at 80 °C. Therefore, other ChCl-based DESs were examined as media of the reaction (Table 1, entries 3–9). Thus, the yields of product A in ChCl:citric acid or in ChCl:lactic acid DES (Table 1, entries 3 and 4) were lower compared to those obtained using the ChCl–urea mixture as the reaction medium. Indeed, although no reaction occurred at room temperature in this system (Table 1, entry 5), increasing the temperature up to 60 °C resulted in very high yields, indicating that the best results was generated with the use of the DES composed of ChCl and urea (molar ratio) (Table 1, entry 6). Noteworthy, increasing the reaction temperature did not change the result in terms of reaction yields (Table 1, entry 7).

As shown in Table 1, it was shown that heating 2-furaldehyde (1 mmol) and morpholine (2 mmol) in the ChCl–urea mixture at 60 °C was considered as the optimum conditions for the synthesis of 4,5-dimorpholinocyclopent-2-enone (**1A**). An important advantage of this solvent system is that the use of ChCl/urea is beneficial for the easy reaction mechanism without using any chromatographic or other purification methods in addition to reduction in reaction times. In fact, compound A was recovered by simple extraction with diethyl ether, followed by separation and removal of the solvent under reduced pressure. A crucial factor in the development of a total full green protocol is the environmental impact of the disposal of huge amounts of industrial solvents, which must match the requirements of the “Green Chemistry Principles” [42]. Therefore, the possibility to use a green solvent not only during the reaction but also during the final extraction of the cyclopentenone derivatives was taken into account. The following green biosolvents [43] were evaluated: ethyl lactate, ethyl acetate, 2-methyltetrahydrofuran, cyclopentyl methyl ether, dimethyl carbonate, diethyl carbonate. The performance of all these media during the extraction of compound A was comparable to diethyl ether. Therefore, taking into account the solvent selection guides that are available [44,45], we decided to develop our protocol choosing ethyl acetate as the “greenest” solvent for the extraction of compound **1A** from the DES.

In a recent report by our group, we observed that using a slight excess of amine (2.2 mmol) with respect to furfural in water, compound **1A** was converted into the thermodynamically stable 2,4-derivative **1B** [20]. As a result, we decided to extend the scope of the present protocol further. Thus, we performed the model reaction heating furfural with 2.2 mmol of morpholine in the ChCl–urea mixture. After 5 min of reaction at 60 °C, compound **1A** was still afforded in 97% (Table 1, entry 8), but increasing the reaction time beyond 4 h, product **1A** was totally converted into **1B** (Table 1, entry 9). The application of microwave irradiation with the aim to provide the enhanced reaction rate of this second process [46,47,48,49,50,51,52,53] failed, as it furnished a mixture of both products **1A** and **1B** even after prolonged reaction time (entry 10, 11). Therefore, the best synthesis of 2,4-dimorpholino cyclopentenone **1B** occurred at 60 °C for four hours with the use of ChCl–urea DES (Table 1, entry 9). These experimental conditions were applied to widely enlarge the application field of the method (Table 2). The reaction gave excellent results for different secondary amines, allowing the isolation of products **1A**–**10A** in high yields (Table 2, entries 1–10). Treatment of furfural with excess of aniline under our reaction conditions did not afford the expected result (Table 2, entry 11) but in that case, the reaction furnished a mixture of the corresponding imine and product **11A** (see Supplementary Materials).

The reaction with primary aliphatic amines, such as benzylamine, produced the corresponding imine as the only product even after prolonged reaction time (Table 2, entry 12). When a slight excess of the appropriate secondary amine was used for a prolonged period of time, the formation of 2,4-bisubstituted cyclopentenone derivatives **1B**–**2B** was detected. The ChCl–urea DES was found to be very effective in this kind of synthesis, since reaction time was not longer than four hours, and purity of final compound, as well as the yields, was satisfied (Table 2, entries 13, 14). As already reported [20], the importance of the nucleophilic character of the functional group was evidenced in this new DES assisted protocol. Accordingly, when cysteine was added to the reaction system, the 4-thio derivative was the major product of the reaction (Table 2, entry 15).

**Scheme 1:**
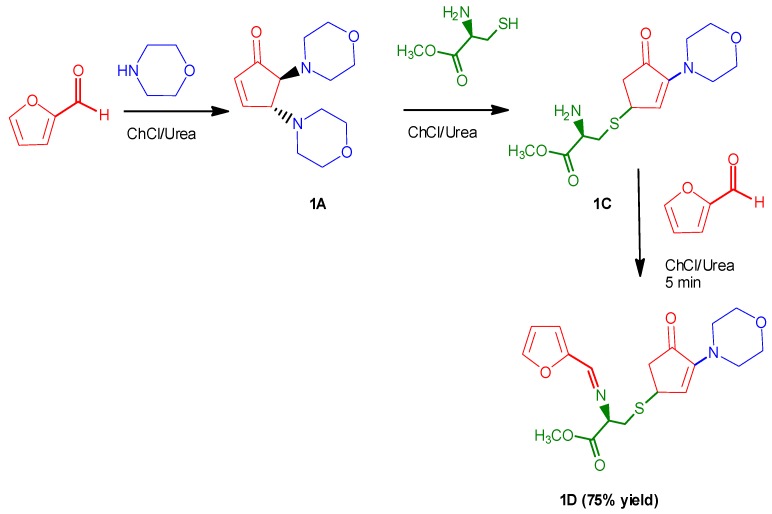
One-pot synthesis of cyclopentenone derivative **1D**.

The observed ability of DES to rapidly form the imine functionality when furfural is treated with a primary amine (Table 2, entries 11, 12) prompted us to exploit the reactivity of the free primary amino group in compound **1C**. On the other hand, the synthesis of Schiff bases in DESs is also a well-known reaction [54,55,56,57,58]. Moreover, the protection and/or modification of the amino function of amino acids [59,60,61,62,63] are one of the most important issues in the synthesis of biologically active compounds and particularly in the synthesis of peptidomimetics [64,65,66,67,68,69]. For this aim, after the formation of **1C** in DES, the simple addition of 1 mmol of furfural to the reaction mixture furnished the corresponding imine **1D** in a one-pot protocol (Scheme 1). The entire process afforded a compound with potential pharmacological interest in excellent yields and short reaction time.

The reusability of green solvents, such as ionic liquids (ILs) and DES, is always an advantage for viable commercial and industrial processes, so we have evaluated the recyclability of the DESs in the reaction of furfural with morpholine. The reaction mixture, including ChCl–urea and products, was extracted with ethyl acetate. This extractive procedure was repeated two more times, and the combined organic extracts were washed with water, dried, filtered and evaporated under reduced pressure to afford the crude reaction product. The residual volatile organic solvent present in the DES phase was removed under vacuum evaporation. Then, the next reaction cycle was performed by simply adding new fresh reagents to the same DES. This reaction mixture was again subjected to the above-described procedure, and further reaction cycles were repeated using the recycled DES phase.

**Figure 2:**
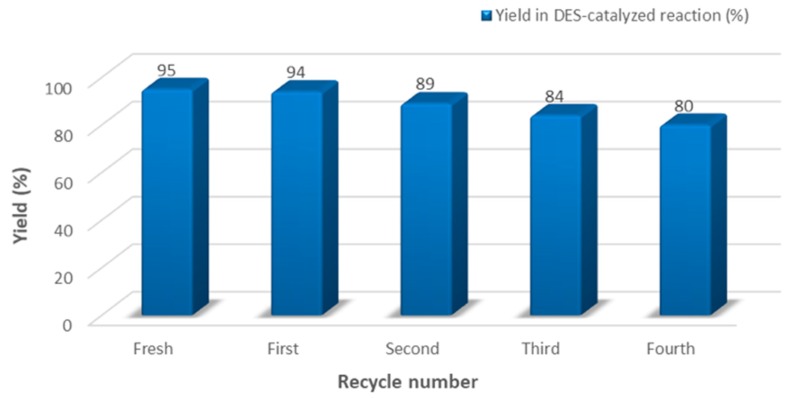
Recyclability of DES. Isolated yields from reaction in the ChCl–urea mixture at 60 °C.

The ChCl–urea DES could be recovered and reused for four consecutive cycles in the synthesis of A obtaining high yields (Figure 2). A slight decrease observed in the last cycle might be due to a modification of the initial DES structure, thus reducing the initial beneficial solvent effects.

Then, in order to demonstrate the potential practical applicability of this green procedure, the model reaction to afford **1A** was carried out in a scale of 10 mmol using 10 mL of DES. The reaction was completed in 30 min with an 87% isolated yield after simple water addition and extraction with ethyl acetate.

**Figure 3:**
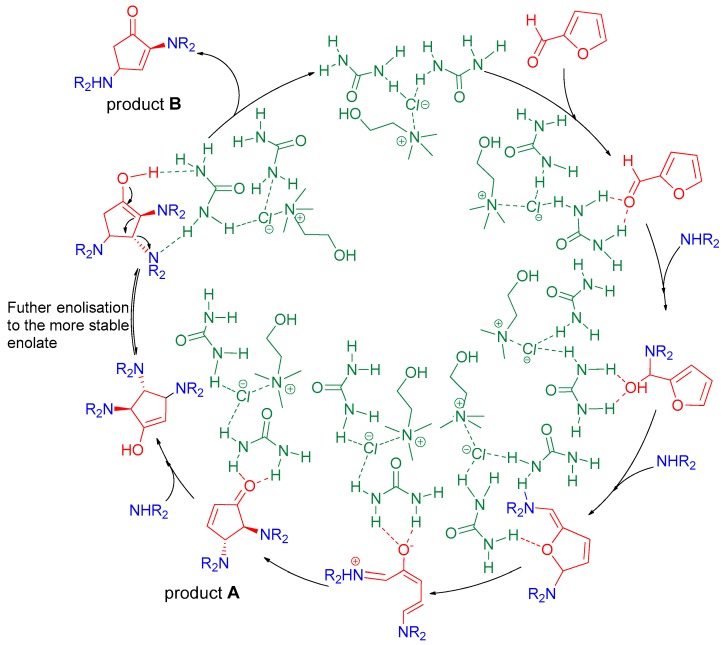
Proposed mechanism of the reaction in DES.

In Figure 3, we tentatively proposed the mechanism of the reaction. Hydrogen bonding is the main factor that influences the reactivity and selectivity of the process [70,71,72,73]. The reversible hydrogen bonding between urea and carbonyl groups giving substrate–solvent complex and activated aldehyde are shown. The initial condensation of activated carbonyl group with the secondary amine in the DES leads to the formation of an enamine intermediate with the loss of a water molecule. Then, this intermediate via a 4π-electrocyclic rearrangement furnishes compound **A** [15,20]. The hydrogen-bond donating character of urea is also involved in the formation of an enolic intermediate that successively leads to the thermodynamically stable regioisomer **B**.

## 3. Materials and Methods 

### 3.1. General Information

All chemicals and solvents were purchased from common commercial sources and were used as received without any further purification. All reactions were monitored by GC/MS analysis (Shimadzu, Kyoto, Japan) and thin-layer chromatography TLC on silica Merck 60 F_254_ pre-coated aluminum plates and were developed by spraying with sulphuric acid in ethanol solution when possible. The GC-MS Shimadzu workstation was constituted by a GC 2010 (equipped with a 30 m-QUADREX 007-5MS capillary column, operating in the “split” mode, 1 mL min-1 flow of He as carrier gas) and a 2010 quadrupole mass-detector. Proton nuclear magnetic resonance (^1^H-NMR) spectra were recorded on a Brüker spectrometer (Bruker Instrument, Inc., Zurich, Switzerland) at 300 MHz. Chemical shifts were reported in δ units (ppm) with tetramethylsilane (TMS) as a reference (δ 0.00). All coupling constants (*J*) were reported in Hertz. Multiplicity was indicated by one or more of the following: s (singlet), d (doublet), t (triplet), q (quartet), m (multiplet). Carbon nuclear magnetic resonance (^13^C-NMR) spectra were recorded on a Brüker at 75 MHz. Chemical shifts were reported in δ units (ppm) relative to CDCl_3_ (δ 77.0). LC-MS analysis were carried using an Agilent 6540 UHD Accurate Mass Q-TOF LC–MS (Agilent, Santa Clara, CA, USA) fitted with an electrospray ionization source (Dual AJS ESI) operating in the positive ion mode. Chromatographic separation was achieved using a C18 RP analytical column (Poroshell 120, SB-C18, 50 × 2.1 mm, 2.7 μm) at 30 °C with an elution gradient from 5% to 95% of B over 13 min, A being H_2_O (0.1% FA) and B CH_3_CN (0.1% FA). The flow rate was 0.4 mL/min. 

### 3.2. General Procedure for the Deep Eutectic Solvent Preparation

A mixture of ChCl (6.98 g, 50 mmol) and urea (6.00 g, 100 mmol) was added in a round-bottom flask under inert atmosphere. The mixture was magnetically stirred for 60 min at 80 °C until a clear colourless liquid was obtained. The obtained DES was used without purification. 

### 3.3. General Procedure for the Formation of 4,5-Difunctionalized Cyclopentenones (***1A**–**12A***)

The amine (1–12, 2 mmol) was added to a stirred solution of furfural (1 mmol) in ChCl–urea eutectic mixture (1 mL). The resulting mixture was stirred at 60 °C for 5 min. The reaction was monitored by TLC and GC/MS analysis. After this time, the reaction was quenched with water and extracted with AcOEt (3 × 2 mL). The organic phases were dried over Na_2_SO_4_, followed by evaporation under reduced pressure to remove the solvent to give the corresponding products **1A**–**12A**. Spectral data were in accordance with the literature [10].

The reaction of morpholine with furfural was scaled up to grams using 10 mL of DES. After completion of the reaction, 10 mL of water was added. The resulting mixture was stirred for 30 min, and after that, the obtained solution was extracted with ethyl acetate (3 × 10 mL). Product **1A** was afforded with a yield of 87%.

### 3.4. General Procedure for the Formation of 2,4-Bifunctionalized Cyclopentenones (***1B**–**2B***) 

The amine (1–2, 2.2 mmol) was added to a stirred solution of furfural (1 mmol) in ChCl–urea eutectic mixture (1 mL). The resulting mixture was stirred at 60 °C for 4 h. The reaction was monitored by TLC and GC/MS analysis. After this time, the reaction was quenched with water and extracted with AcOEt (3 × 5 mL). The organic phases were dried over Na_2_SO_4_, followed by evaporation under reduced pressure to remove the solvent to give the corresponding products **1B**–**2B**. Spectral data were in accordance with the literature [14]. 

### 3.5. Procedure for the Formation of Methyl (2R)-2-(furan-2- ylmethylene) amino)-3-((3-morpholino-4-oxocyclopent-2-en-1-yl)thio]propanoate (***1D***)

Morpholine (2.2 mmol) was added to a stirred solution of furfural (1 mmol) in ChCl–urea eutectic mixture (1 mL). The resulting mixture was stirred at 60 °C for 5 min. After this time, cysteine methyl ester hydrochloride (1 mmol) was added to the reaction mixture and stirred for further 4 h to obtain 4-(methyl-l-cysteinate)-2-morpholino cyclopent-2-enone **1C** [14]. In order to obtain the imine derivative of 1C, 1 mmol of furfural was added to the reaction mixture described above and maintained under stirring for 5 min and monitored by GC/MS analysis. The reaction was quenched with water and extracted with AcOEt (3 × 2 mL). The organic phases were dried over Na_2_SO_4_, followed by evaporation under reduced pressure to remove the solvent to give the corresponding product **1D** in 75% yield. 

^1^H-NMR (300 MHz, CDCl_3_): 2.47 (dd, 1H, *J* = 8.4 Hz, *J* = 4.5 Hz, COCHN=), 2.56 (m, 1H, SCH_2_), 2.85 (m, 1H, SCH_2_ ), 3.13–3.16 (m, 4H, morpholine), 3.44 (dd, 1H, *J* = 8.1 Hz, *J* = 3.3 Hz, COCH_2_), 3.50 (dd, 1H, *J* = 5.1 Hz, *J* = 3.0 Hz, COCH_2_), 3.03 (s, 3H, C*H*_3_), 3.63 (t, 1H, SCH), 3.71–3.82 (m, 4H, morpholine), 6.23 (*d*, *J* = 3.0 Hz, 1H, COC=C*H*), 6.27 (dd, *J* = 3.0 Hz, *J* = 2.1 Hz, 1H, OCH=C*H*), 6.31 (*d*, *J* = 2.1 Hz, 1H, OC=C*H*) 6.38 (*d*, *J* = 3.0 Hz, 1H, OCH=C), 7.33 (s, 1H, C*H*=N); ^13^C-NMR (75 MHz, CDCl_3_) 29.7, 30.4, 44.2, 47.3, 53.1, 66.5, 78.6, 108.9, 110.0, 129.2, 141.8, 142.4, 151.6, 152.9, 174.9, 201.8. MS(EI): *m/z* (%) = 378 (4) [M]^+.^, 319 (3), 277 (2), 245 (3), 212 (18), 180 (100), 166 (47), 146 (4), 138 (13), 120 (16), 111 (17), 93 (13), 44(8). HRMS (ESI) for ([C_18_H_22_N_2_O_5_S] + H)^+^ 378.1249, found 379.1328 [M + H]^+^, 401.1143 [M + Na]^+^. 

### 3.6. Recycling of Deep Eutectic Solvent

The recycling of DES was examined in the reaction of furfural and morpholine in the ChCl–urea mixture under optimized conditions. After the reaction was completed, AcOEt (3 mL) was added to the reaction mixture, and stirred for 5 min. The stirring was stopped to allow phase separation, and the upper organic layer was removed. This extractive procedure was repeated two more times and the combined organic extracts were washed with water (3 × 5 mL), dried (Na_2_SO_4_), filtered, and evaporated under reduced pressure to afford the crude reaction product. The residual volatile organic solvent present in the DES phase was removed under vacuum evaporation. Then, the next reaction cycle was performed with the DES, adding fresh furfural and morpholine. This reaction mixture was again subjected to the above-described procedure, and further reaction cycles were repeated using the recycled DES phase. 

## 4. Conclusions

In conclusion, we have described an effective and rapid method for the synthesis of bifunctionalized cyclopentenones in an eco-friendly and biodegradable DES based on the ChCl–urea mixture. The advantages of this method are operational simplicity, green medium, DES recoverability, short reaction time, high yields and no chromatographic purification procedures. The recyclability of the DES (up to four reaction runs) and its synergistic effect on the reaction outcome have also been demonstrated. Furthermore, the DES can be used as a low-cost, safe and efficient solvent with feasible large-scale preparation.

To the best of our knowledge, this is the first report on the synthesis of *trans*-4,5-diaminocyclopent-2-enones derivatives and 2,4-diaminocyclopent-2-enones in DESs.

Moreover, this synthetic strategy can be utilized for the construction of many other novel cyclopentenone derivative compounds of pharmaceutical importance.

## Figures and Tables

**Table 1:**
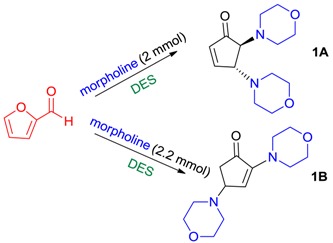
Optimization of the reaction conditions in model reaction ^1,2^.

Entry	DES (Molar Ratio)	Temp (°C)	Time (min)	Product	Yield ^3^ (%)
1 ^1^	ChCl:glycerol (1:2)	rt	60	**1A**	-
2 ^1^	ChCl:glycerol (1:2)	80	5	**1A**	20
3 ^1^	ChCl:citric acid (1:1)	80	5	**1A**	35
4 ^1^	ChCl:lactic acid (1:1)	80	5	**1A**	45
5 ^1^	ChCl:urea (1:2)	rt	60	**1A**	-
6 ^1^	ChCl:urea (1:2)	60	5	**1A**	95
7 ^1^	ChCl:urea (1:2)	80	5	**1A**	95
8 ^2^	ChCl:urea (1:2)	60	5	**1B**	0 ^4^
9 ^2^	ChCl:urea (1:2)	60	240	**1B**	98
10 ^2^	ChCl:urea (1:2)	60 (MW)	30	**1B**	45 ^5^
11 ^2^	ChCl:urea (1:2)	60 (MW)	60	**1B**	45 ^5^

^1^ General reaction conditions for the synthesis of *trans*-4,5-dimorpholinocyclopent-2-enone **1A**: furfural (1 mmol) and morpholine (2 mmol) are dissolved in a DES and stirred for 5–60 min at different temperatures. ^2^ General reaction conditions for the synthesis of 2,4-dimorpholinocyclopent-2-enone **1B**: furfural (1 mmol) and morpholine (2.2 mmol) were dissolved in a DES and stirred for 5–240 min at different temperatures. ^3^ Yield (%) calculated from GC/MS data of conversion of furfural. ^4^ Compound **1A** is obtained as the only product (97% yield). ^5^ GC/MS analysis shows the formation of both product **1A** (55% yield) and product **1B** (45% yield).

**Table 2:**
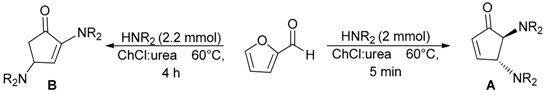
Synthesis of bifunctionalized cyclopentenones from furfural ^a^.

**Entry**	**Amine**	**Products**		**Yields ^b^ (%)**
1	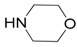	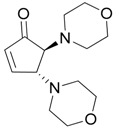	**1A**	9595
2	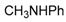	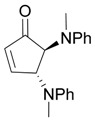	**2A**	90
3	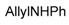	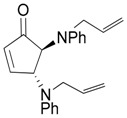	**3A**	92
4	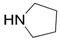	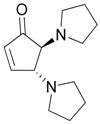	**4A**	95
5	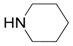	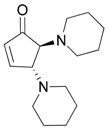	**5A**	96
6	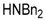	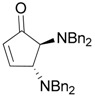	**6A**	89
7	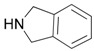	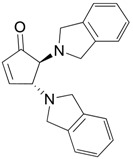	**7A**	91
8	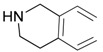	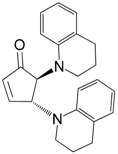	**8A**	93
9	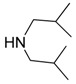	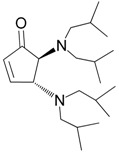	**9A**	94
10	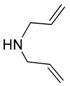	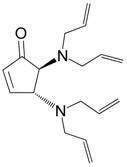	**10A**	94
11	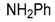	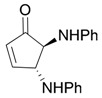	**11A**	40 ^c^
12	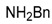	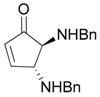	**12A**	0 ^d^
13	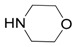	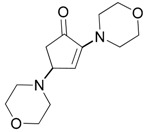	**1B**	98
14	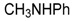	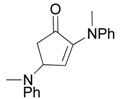	**2B**	94
15	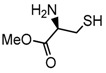	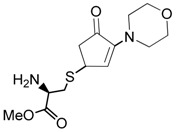	**1C**	85

^a^ General reaction conditions: 1 mmol of furfural and 2 mmol/2.2 mmol of amine are dissolved under stirring in the DES (1 mL) at 60 °C for 5 min/4 h to afford the corresponding products **A**/**B**. ^b^ Yield (%) calculated from GC/MS data. ^c^ The corresponding imine derivative is afforded in 60% yield. ^d^ The corresponding imine is recovered as the sole product.

## Supplementary Materials

The HR-MS, GC/MS, 1H- and 13C-NMR spectra of new synthesized compounds are available online.
