# Supplementary material for: Biorenewable Deep Eutectic Solvent for Selective and Scalable Conversion of Furfural into Cyclopentenone Derivatives
Source: Molecules. 2018 Jul 28;23(8):1891. doi: 10.3390/molecules23081891 (PMC6222497; doi:10.3390/molecules23081891)

Biorenewable Deep Eutectic Solvent for Selective and Scalable Conversion of Furfural into Cyclopentenone Derivatives

Maria Luisa Di Gioia,[†] *Monica Nardi,*[*§,‡] *Paola Costanzo,*[‖] *Antonio De Nino,*[§] *Loredana Maiuolo,*[§] *Manuela Oliverio,*[‖] *and Antonio Procopio*[‖]

[†] Dipartimento di Farmacia e Scienze della Salute e della Nutrizione, Edificio Polifunzionale, Università della Calabria, 87036 Arcavacata di Rende, Cosenza.

[§] Dipartimento di Chimica, Università della Calabria, Cubo 12C, 87036-Arcavacata di Rende (CS), Italy, Tel.: +39 0984 492850. Fax: +39 0984 493307. E-mail: monica.nardi@unical.it

[‡] Dipartimento di Agraria, Università Telematica San Raffaele, Roma, Via di Val Cannuta, 247, 00166, Italia.

[‖] Dipartimento di Scienze della Salute, Università Magna Græcia, Viale Europa, 88100-Germaneto (CZ), Italy

Supporting Informations

LIST OF CONTENTS

GC/MS analysis (EI spectrum) - Sample **1B**

Spectroscopic analysis of compound **1D**

HRMS-Spectrum - Sample **1D**

GC/MS analysis (EI spectrum) - Sample **1D**

^{1}H-NMR-Spectrum - Sample **1D**

^{13}C-NMR -Spectrum - Sample **1D**

GC/MS analysis (EI spectrum) of the reaction between furfural and aniline

GC/MS analysis (EI spectrum) – Sample ***N*-(furan-2-ylmethylene)aniline**

GC/MS analysis (EI spectrum) compound **1B**

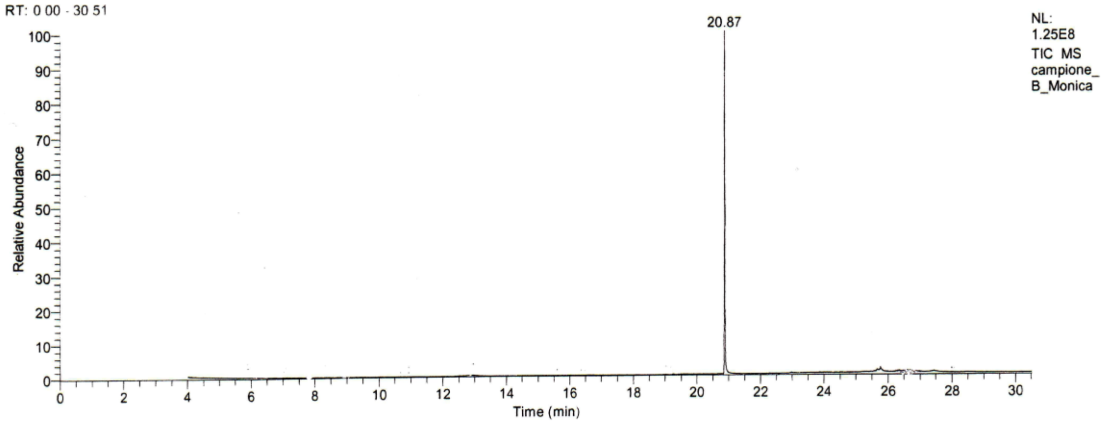

RT	Peak Area	Peak Height	Area %	S/N
26.70	22473900.70	4086859.57	11.10	1734.53
20.87	179973257.78	120805596.68	88.90	51271.80

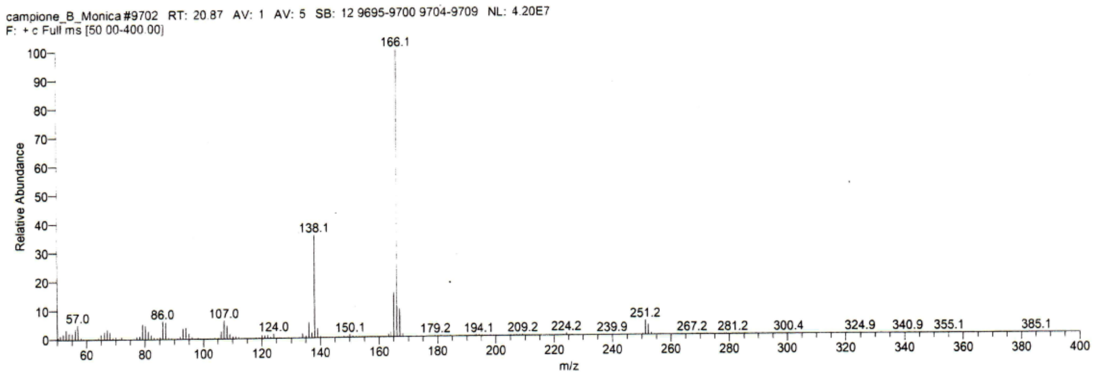

Spectroscopic analysis of compound **1D**

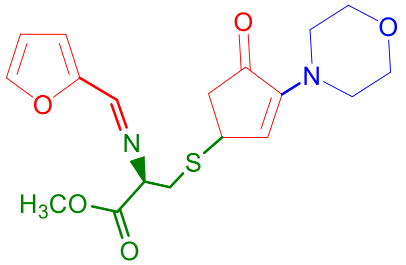

HRMS analysis

**Sample Type**	Sample	**Position**	Vial 91
**Instrument Name**	Instrument 1	**User Name**	
**Acq Method**	bocpep.m	**Acquired Time**	5/18/2017 12:30:40 PM
**IRM Calibration Status**	Success	**DA Method**	Default.m
**Comment**			

**Sample Group**		**Info.**
**Acquisition SW Version**	6200 series TOF/6500 series Q-TOF B.05.01 (B5125)	

Compound Table

Compound Label	RT	Mass	Abund	Formula	Tgt Mass	Diff (ppm)	MFG Formula	DB Formula
Cpd 1: C18 H22 N2 O5 S	4,814	378,1215	877322	C18 H22 N2 O5 S	378,1249	-9,1	C18 H22 N2 O5 S	C18 H22 N2 O5 S

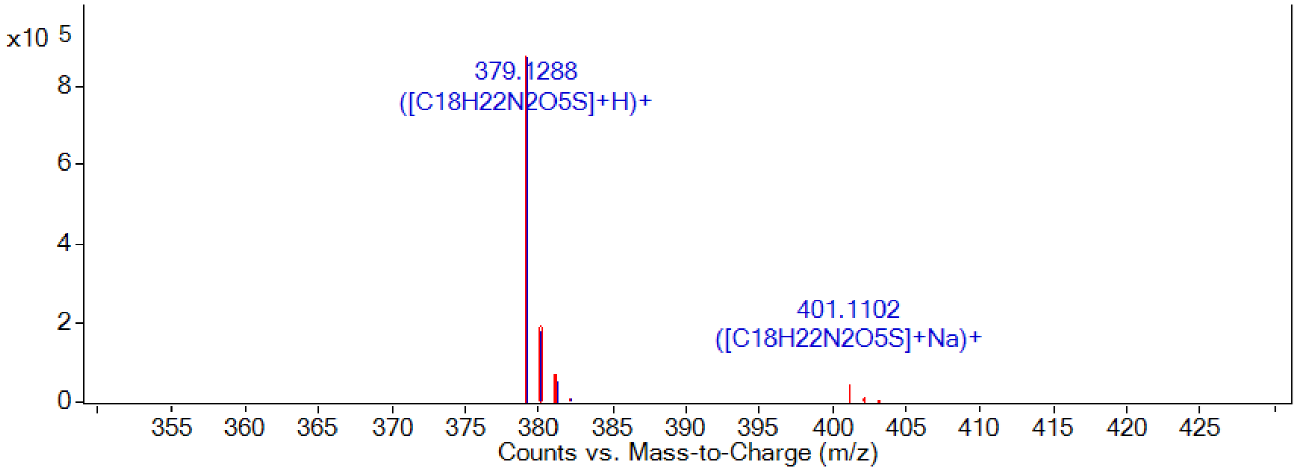

MS Spectrum Peak List

m/z	z	Abund	Formula	Ion
379,1288	1	877322,31	C18H22N2O5S	(M+H)+
380,1318	1	174157,52	C18H22N2O5S	(M+H)+
381,1289	1	54203,96	C18H22N2O5S	(M+H)+
382,1255	1	9722,57	C18H22N2O5S	(M+H)+
401,1102	1	39357,7	C18H22N2O5S	(M+Na)+
402,1129	1	9052,84	C18H22N2O5S	(M+Na)+
403,1135	1	3030,66	C18H22N2O5S	(M+Na)+

GC/MS analysis (EI spectrum)

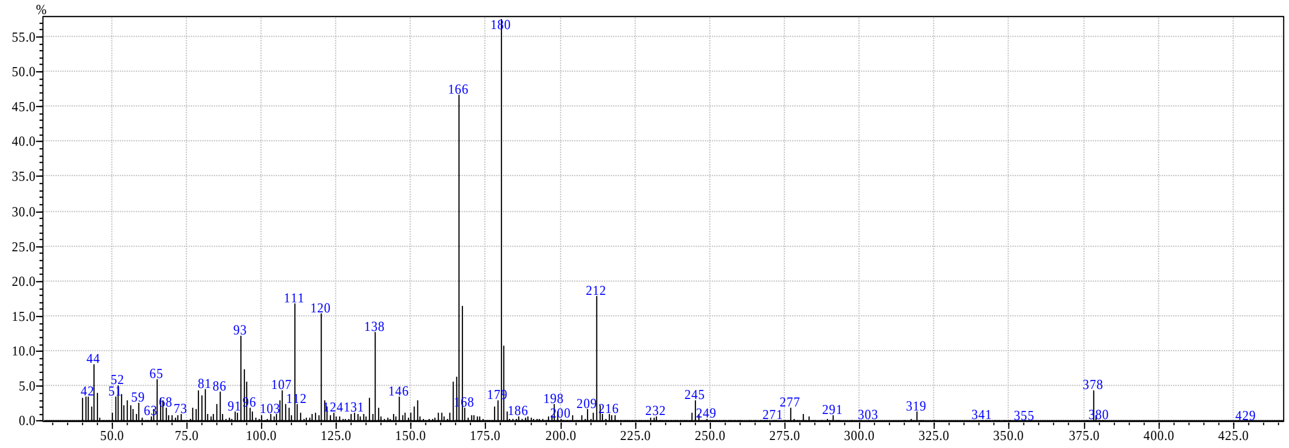

^{1}H-NMR

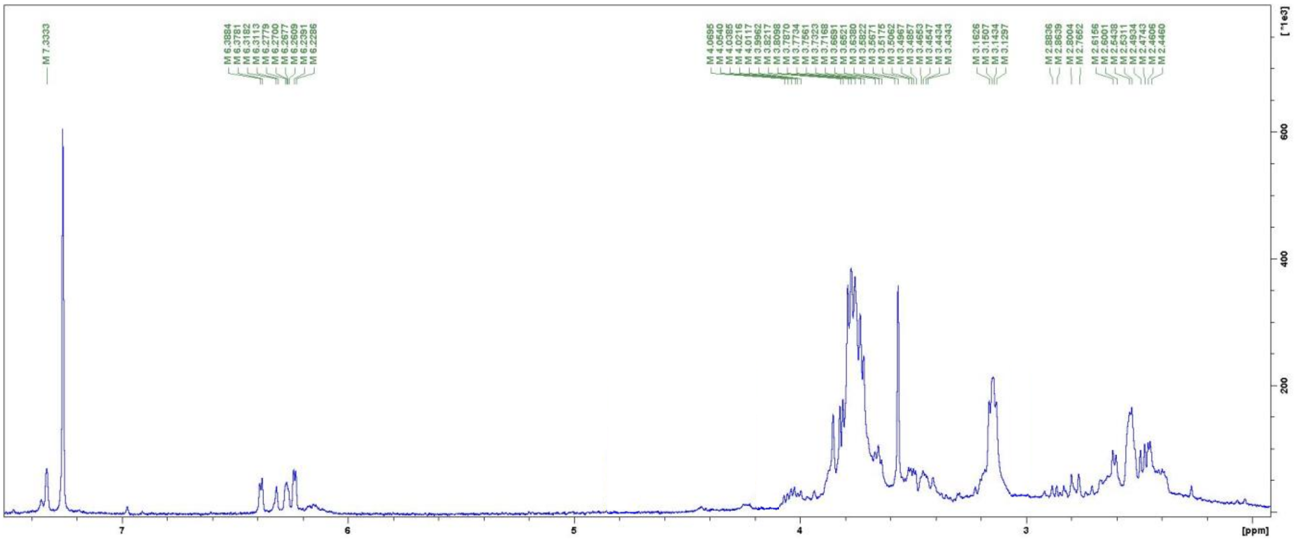

^{13}C-NMR

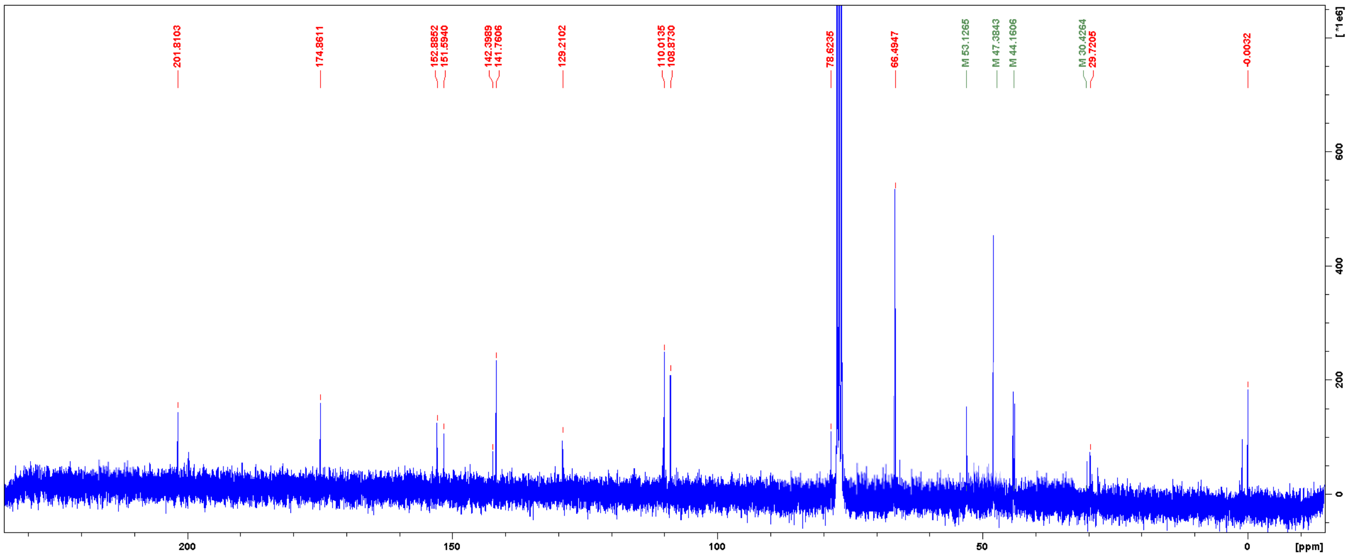

GC/MS analysis (EI spectrum) of the reaction between furfural and aniline :

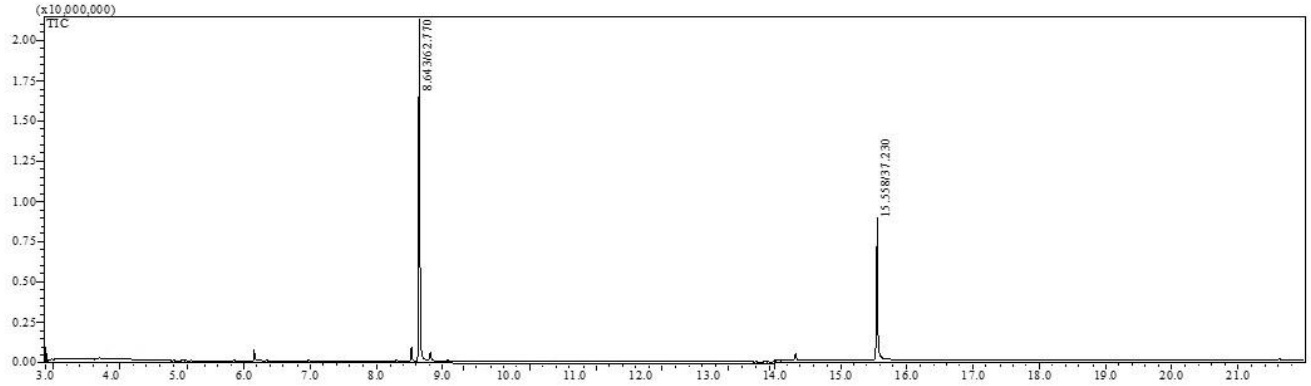

r.t. 8.643 min

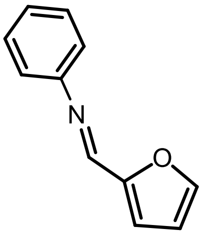

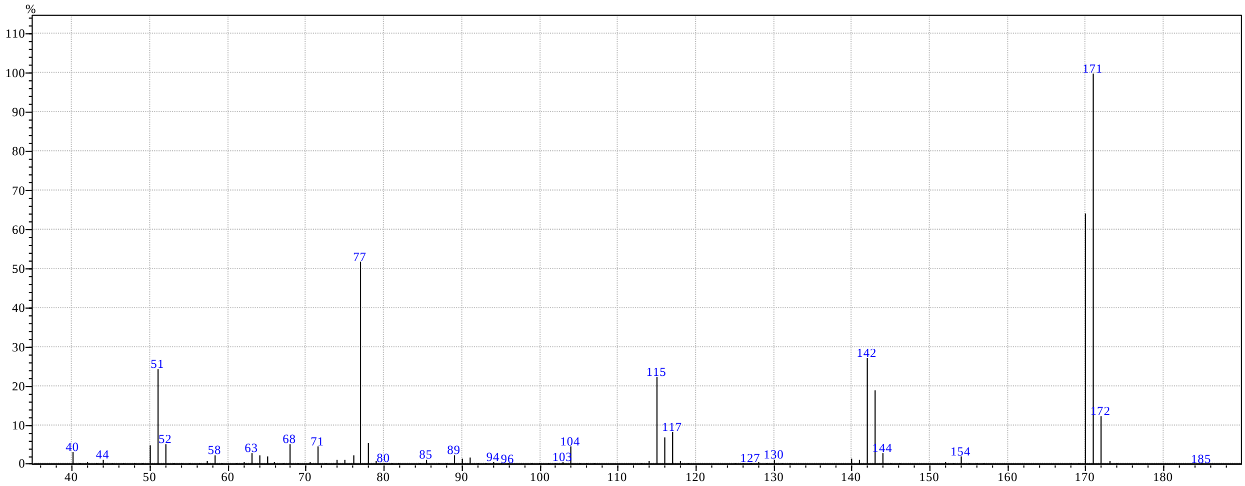

r.t. 15.558 min

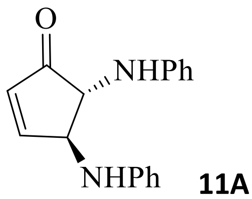

11A

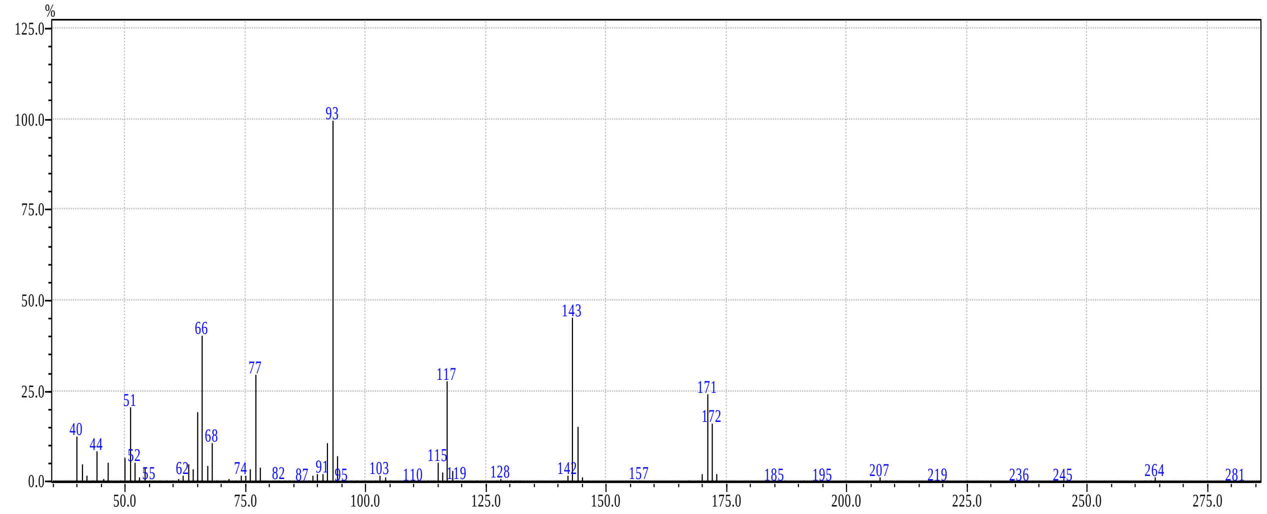

Supplement: Supplementary file 1 [file molecules-23-01891-s001.pdf]
